# Potential of coconut oil as a mosquito repellent

**DOI:** 10.1186/s41182-025-00714-8

**Published:** 2025-04-23

**Authors:** Shiho Hara, Micheal Teron Pillay, Toshihiko Sunahara, Masaru Nagashima, Lucy Atieno Okech, Chiaki Tsurukawa, Yasuhiko Kamiya

**Affiliations:** 1 Tropical Medicine and Global Health, Nagasaki University, Nagasaki, Japan; 2 Department of Vector Ecology and Environment, Institute of Tropical Medicine (NEKKEN), Nagasaki University, Nagasaki, Japan; 3 Institute of Developing Economies Japan External Trade Organization (IDE-JETRO), Tokyo, Japan; 4 Nagasaki Kenya Institution, Homabay, Kenya

**Keywords:** Coconut oil, Natural repellent, Mosquito bite, Vectors

## Abstract

**Background:**

Naturally derived products have become popular as a mosquito repellent in addition to mosquito nets and chemical repellents. Coconut-derived fatty acids have demonstrated repellent properties against various blood-feeding arthropods, including mosquitoes. Daily use moisturizers and body soaps containing coconut have displayed some repellent effect against mosquitoes. However, no studies have been conducted on coconut oil specifically, and the effects of pure coconut oil still remain unknown in the western Kenya region.

**Methods:**

In this study, we investigated the effect of coconut oil on decreasing mosquito bites in a laboratory and field setting. Using *Anopheles stephensi* mosquitoes, the laboratory experiment compared coconut oil treated and non-treated membranes on a Hemotek blood feeding device. In the cross-sectional study in western Kenya, we investigated bite counts among 490 children, 5 years and under. Descriptive analysis, simple, multiple and mixed regression models were employed. The outcome was the number of mosquito bite marks, the primary explanatory variable was skin cream types, in addition to demographic, environmental, behavioral and socio-economic variables.

**Results:**

Coconut oil significantly reduced mosquito blood feeding, with a pooled Mantel–Haenszel odds ratio of 0.06, a Mantel–Haenszel chi-square statistic of 79.82 (*p* = 0.01), and an average blood-feeding rate of 1% compared to 31% in the control group. The mixed model identified significant factors influencing mosquito bite counts while accounting for village-level random effects. Coconut oil users experienced 15% reduction in bites (*p* = 0.01) compared to synthetic creams users. High and medium cream application frequencies reduced bites by 57% (*p* < 0.001) and 17% (*p* = 0.007), respectively. Late cream application and late net entry significantly increased bite counts by 41% (*p* < 0.001) and 53% (*p* < 0.001), respectively. In addition, higher temperatures from the preceding 2 weeks in the region was associated with a 26% (*p* = 0.003) increase in bite counts.

**Conclusions:**

These findings underscore the protective impact of cream application and timing and net use timing, as well as environmental temperature influences on bite outcomes. Particularly, the effect of coconut oil in decreasing mosquito bites and its potential as an alternative repellent has been observed in both laboratory and field settings.

**Supplementary Information:**

The online version contains supplementary material available at 10.1186/s41182-025-00714-8.

## Background

Vector-borne diseases, particularly those transmitted by mosquitoes, pose significant global health challenges. Mosquitoes are efficient vectors due to their adaptability to diverse environments, transmitting diseases, such as malaria, dengue fever, and chikungunya, which cause substantial morbidity and mortality [1, 2]. In 2022, 249 million malaria cases were recorded globally, with Africa bearing 95% of the burden [3]. Children under 5 years particularly accounted for approximately 76% of malaria-related deaths since 2015, despite increased use of bed nets [3]. In Kenya, malaria incidence rates are still high, at 0.19 cases per person per year [4]. This highlights the urgent need for effective prevention strategies against vectors, with some of the easily implemented interventions such as alternative repellents [5] being applied to complement existing methods.

Repellents are substances applied to deter insects from landing or biting and are crucial in preventing diseases transmitted by vectors, such as mosquitoes [6]. They range from synthetic chemicals, such as N,N-Diethyl-meta-toluamide (DEET) and Ethyl butylacetylaminopropionate (IR3535), to plant-based oils [7, 8]. DEET, regarded as the gold standard, has been pivotal in potentially reducing the burden of vector-borne diseases, such as malaria, dengue, and Zika virus [9, 10]. However, natural repellents derived from plant oils are gaining popularity for their safety, cost-effectiveness, and environmental friendliness [11, 12].

In recent years, there has been a growing interest in natural mosquito repellents due to preference for non-chemical ingredients and cost-effectiveness compared to synthetic alternatives [11, 12]. These repellents, often derived from plant extracts and essential oils, have long been used in traditional medicine for their insect-repellent properties [5]. Common examples include citronella, eucalyptus, lemon eucalyptus, lavender, and peppermint oils, which act by forming a vapor layer on the skin that emits a scent intolerable to mosquitoes, thereby preventing bites [14]. Citronella oil, one of the most widely used natural repellents, has been shown to provide short-term protection against mosquitoes [15, 63]. Lemon eucalyptus oil, which contains the active compound p-menthane-3,8-diol, has a comparable effectiveness to low concentrations of DEET and offers longer-lasting protection [16, 19]. Despite their natural origin, plant-based repellents can vary in effectiveness and duration of protection, often requiring more frequent reapplication compared to synthetic repellents [16]. Although natural repellents may require more frequent reapplication due to their shorter duration of effectiveness, they are considered safer for vulnerable groups, such as infants, pregnant women, and individuals with sensitive skin [5]. Their versatility allows for application in various forms, including soaps, creams, and sprays, making them accessible and practical for daily use. Continued research into improved formulations and various unexplored plant-based repellents has offered promising alternatives against mosquito bites, particularly in resource-limited settings where access to chemicals such as DEET is challenging [17, 18]. In Western Kenya, the use of natural oils as moisturizing creams is common, with preference for coconut oil especially for children. Among these, coconut oil-based formulations have shown promise in repelling mosquitoes [13].

Coconut oil is derived from the kernel and meat of mature coconuts and is known for its versatility in cosmetic and medicinal applications [20, 21]. Chemically, it is composed mainly of saturated fats, with lauric acid making up approximately 50% of its content. Other fatty acids present include myristic, palmitic, and caprylic acids [19, 22]. The benefits of coconut oil are indeed wide-ranging with the fatty acids in coconut oil have been shown experimentally to repel blood-feeding arthropods, flies, ticks, bed bugs, and even mosquitoes, such as *Aedes aegypti* [13]. Previous studies [5, 8, 13, 24–26] have shown that daily use moisturizers and body soaps containing coconut fatty acids and coconut oil have a repellent effect against mosquitoes, offering a promising alternative to synthetic chemicals [13, 19]. However, no studies have been conducted on coconut oil alone, and the effects of coconut oil in a natural setting remain unknown.

This study investigates the efficacy of coconut oil as a mosquito repellent in the Lake Victoria region of western Kenya, an area with the highest malaria incidence in the country due in part to the abundance of *Anopheles* mosquitoes [27, 28]. Despite government efforts to promote mosquito net use and other control measures, malaria remains a significant public health challenge [29]. While commercial repellents are effective against mosquito bites, their high cost and limited accessibility in rural areas leave vulnerable populations, such as children and the elderly, at heightened risk [4, 27]. For instance, DEET costs as much as ten times the retail price of coconut oil in Kenya, with DEET-based insect repellents generally only available in urban areas of Kenya, while they are not easily found in rural regions [74].

Given the need for affordable and accessible alternatives, coconut oil emerges as a promising candidate. In the Lake Victoria area, moisturizing creams are commonly used by people, and coconut oil is a familiar product. According to the Kenya Agricultural & Livestock Research Organization [60], 99% of certain types of coconuts are cultivated along the Kenyan coast, and the monetary value of coconuts and coconut products is approximately 3.2 billion Kenyan Shillings. This makes coconut oil an affordable option compared to commercial repellents.

This study specifically aims to determine the effect of coconut oil-based cream use on (1) the number of blood-feeding mosquitoes in laboratory experiments to establish feasibility and test protocols before fieldwork commenced, and (2) mosquito biting frequency in children 5 years and under within Homa Bay, Kenya. By evaluating these aspects, the study will provide a holistic understanding of the factors influencing the efficacy of coconut oil. If proven effective, coconut oil could serve as a cost-effective intervention, particularly during peak mosquito activity, contributing to malaria prevention and control efforts in resource-limited settings.

## Methods

### Study site

**Fig. 1:**
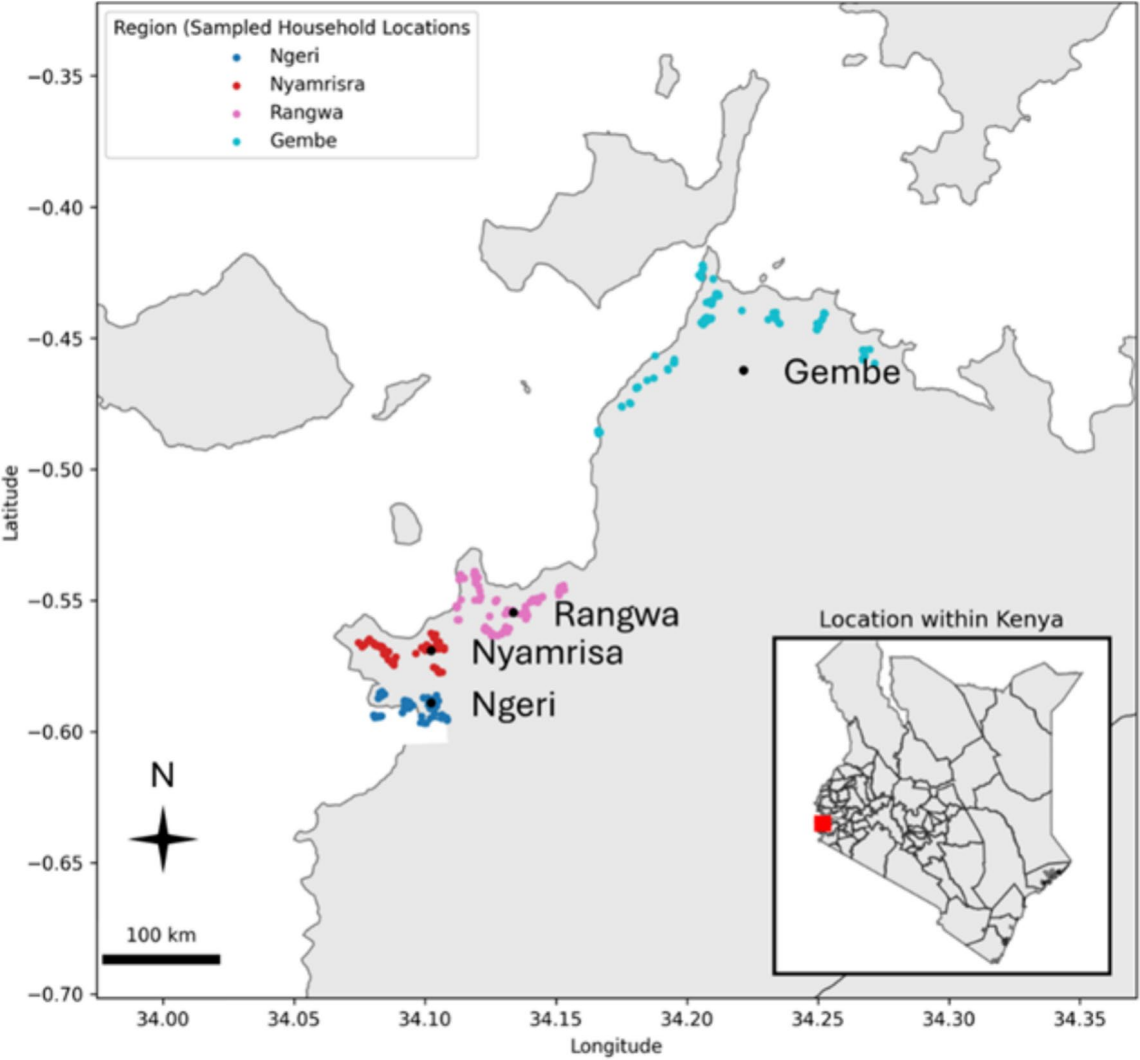
Study site map of West Homa Bay County with colored dots (blue, red, pink and light blue for Ngeri, Nyamrisa, Rangwa and Gembe areas, respectively) indicating the households sampled in the survey

Kenya, located in East Africa has a climate which varies from tropical along the coast to arid in the interior, with coastal regions experiencing hot, humid conditions and highland areas characterized by moderate rainfall throughout the year. The field study was conducted in Homa Bay County, which lies in the western part of Kenya and borders Lake Victoria (Fig. 1). The region spans approximately 3154.7 square kilometers and includes administrative sub-counties (Suba South and Mbita). The climate in Homa Bay County is warm and temperate, with average annual temperatures ranging from 18 °C to 28 °C and rainfall averaging about 1200 mm with the peak rainy season occurring from March to June [31]. However, the region is increasingly affected by climate change, with issues such as flooding and soil erosion becoming more common [30]. Homa Bay’s economy relies heavily on fishing, subsistence farming, and livestock herding [32, 33].

### Laboratory experiment

Laboratory experiments were conducted at Institute of Tropical Medicine, Nagasaki University, Japan. The experiments aimed to evaluate the effectiveness of coconut oil in preventing mosquito blood-feeding.

**Fig. 2:**
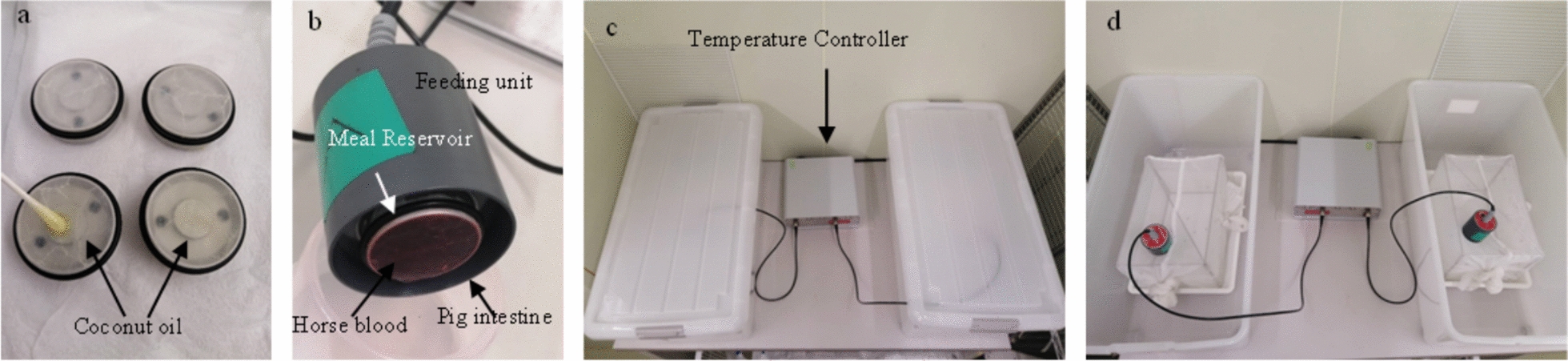
Picture of meal reservoir with membrane. **a** Upper reservoir contains no oil, while the lower reservoir is treated with coconut oil. **b** Hemotek membrane feeding system. This system includes a feeding unit featuring a meal reservoir with a horse blood sample and a pig intestine membrane. **c**, **d** Two feeding units placed on each mosquito cage in the cases and connected to temperature controller

*Anopheles stephensi* mosquitoes were obtained from the National Institute of Infectious Diseases, Tokyo, Japan. These mosquitoes were reared in a controlled environmental chamber at 25 °C, 70% humidity, and a 14 h light cycle. To account for time constraints, the mosquitoes were acclimatized to daytime feeding schedules using a growth chamber (MLR-352H、Panasonic Co. Tokyo, Japan) 2–3 days prior to the experiments (Fig. 2). The mosquitoes were fed with a 3% sugar solution duration for on 10 days [36]. Before the blood feeding experiments, the mosquitoes were not fed for around 12 h. Mosquitoes were approximately 10 days and had not been provided blood meals before the trials [35, 36]. This period of 10 days was chosen based on existing blood feeding experimental protocols [34, 36], as female *anopheles* blood feeding is more consistent once matured, which is around 7–10 days and host seeking behavior is usually stronger and more consistent in mosquitoes over 5 days [71]. Each cage contained 30 mosquitoes to facilitate accurate counting. The experiments were conducted using a Hemotek artificial membrane feeding system (Hemotek Ltd., Blackburn, UK) designed to simulate human skin. Pig intestine membranes were used as a substitute for skin, while horse blood replaced human blood based on established protocols [36]. Membranes were cleaned with distilled water, cut to size, and secured to the meal reservoir with rubber rings. The feeding units were warmed and placed on top of mosquito cages, with the membranes directly contacting the cage netting. Mosquitoes were allowed to feed for 1 h. Afterward, all female mosquitoes were crushed to count the number of blood-fed individuals. Control (no oil) and intervention (coconut oil applied to membranes) groups were tested alternately to minimize bias. Membranes and feeding trays were cleaned with boiling water after each trial to eliminate residual odors.

### Field survey

The field study targeted children under 5 years in Homa Bay County, a high-risk group for malaria. Households were selected using stratified random sampling. Clusters from a previous Science and Technology Research Partnership for Sustainable Development (SATREPS) project [72], each comprising 20 nearby households, served as the basis for sampling in three regions. An additional region, Gembe West, was sampled using simple random sampling. All selected households had children under five, ensuring consistency in the sampling criteria. The final sample size was 522 participants from 329 households.

**Fig. 3:**
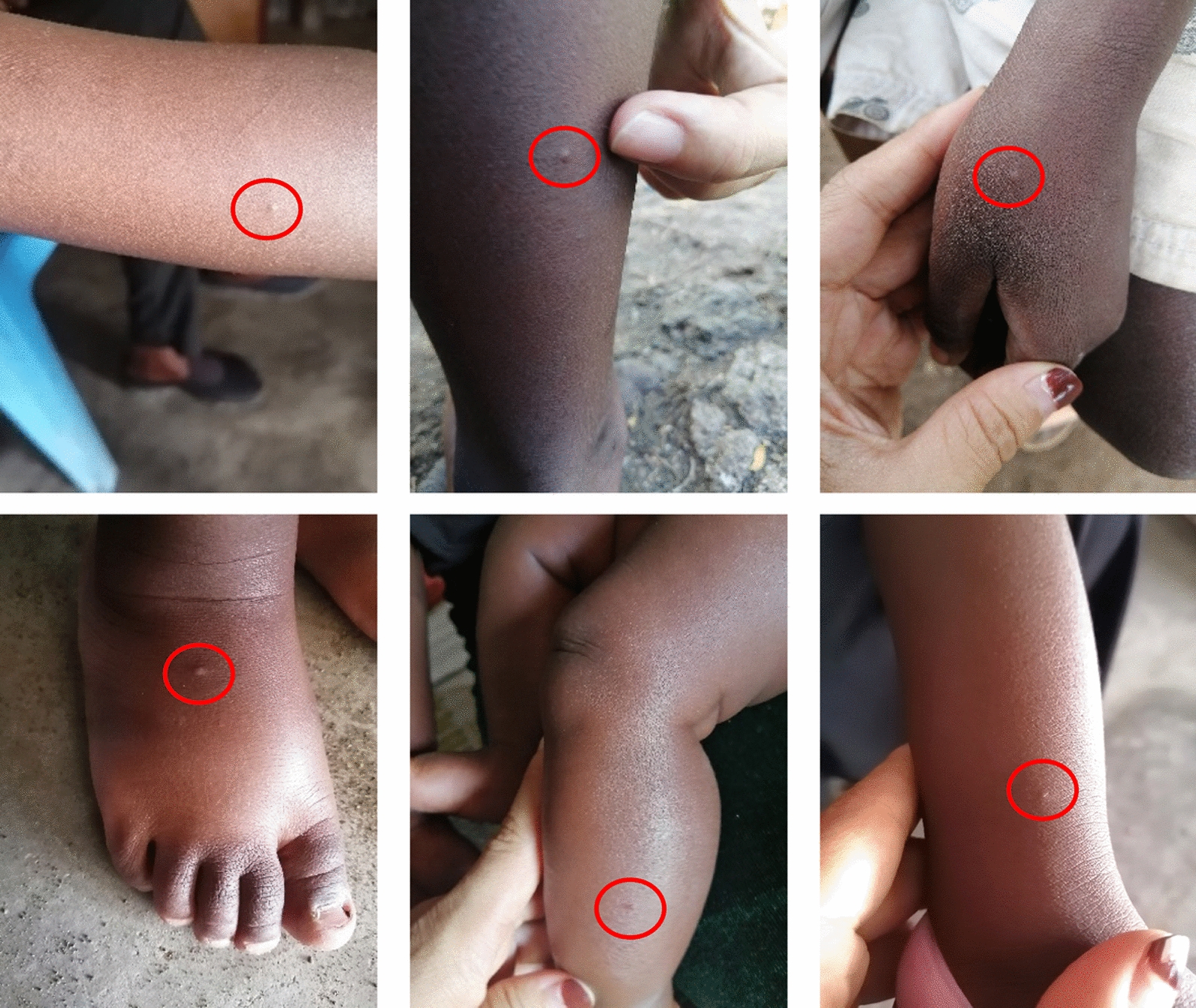
Mosquito bite marks. Shape, size and singular marks were identified as a single bite count

A standardized definition of mosquito bite marks was developed through preliminary observations of 100 children at a local pediatric clinic (Fig. 3). Marks were identified as isolated swelling or welts protruding from the skin. Cases with multiple swellings or rashes, suggestive of other skin conditions, were excluded [37, 38]. Observations were made at a single timepoint for each participant. Data collection also involved administering questionnaires to parents/guardians (see Appendix S1). To minimize bias, questions about moisturizers were asked before bite counts were recorded. Bite marks were photographed for verification, and moisturizer containers were inspected to identify the brands, type and ingredients. The temperature and rainfall data were extracted with a 2 week lag for each user based on latitude, longitude and the date of interview as this lag is commonly indicated as high risk for malaria incidence and can be a good proxy for mosquito abundance [43].

### Data analysis

The data for blood feeding and non-feeding mosquitoes across the control and coconut treated groups were analyzed using odds ratio for each individual experiment. This was done to quantify the effect of coconut oil use on mosquito blood feeding. The odds ratio was specifically chosen to account for the outcome (blood feeding), being a rare occurrence especially in the coconut treated group [62]. A continuity correction was applied to address zero counts in some contingency tables to avoid division by zero errors [39]. Fisher’s exact test assessed the association between treatment type and blood feeding, and the Mantel–Haenszel test was used to pool ORs’ across experiments while adjusting for variability between trials. The Mantel–Haenszel chi-squared test confirmed associations between treatment type and blood feeding status, also adjusting for experimental repetitions [40].

**Fig. 4:**
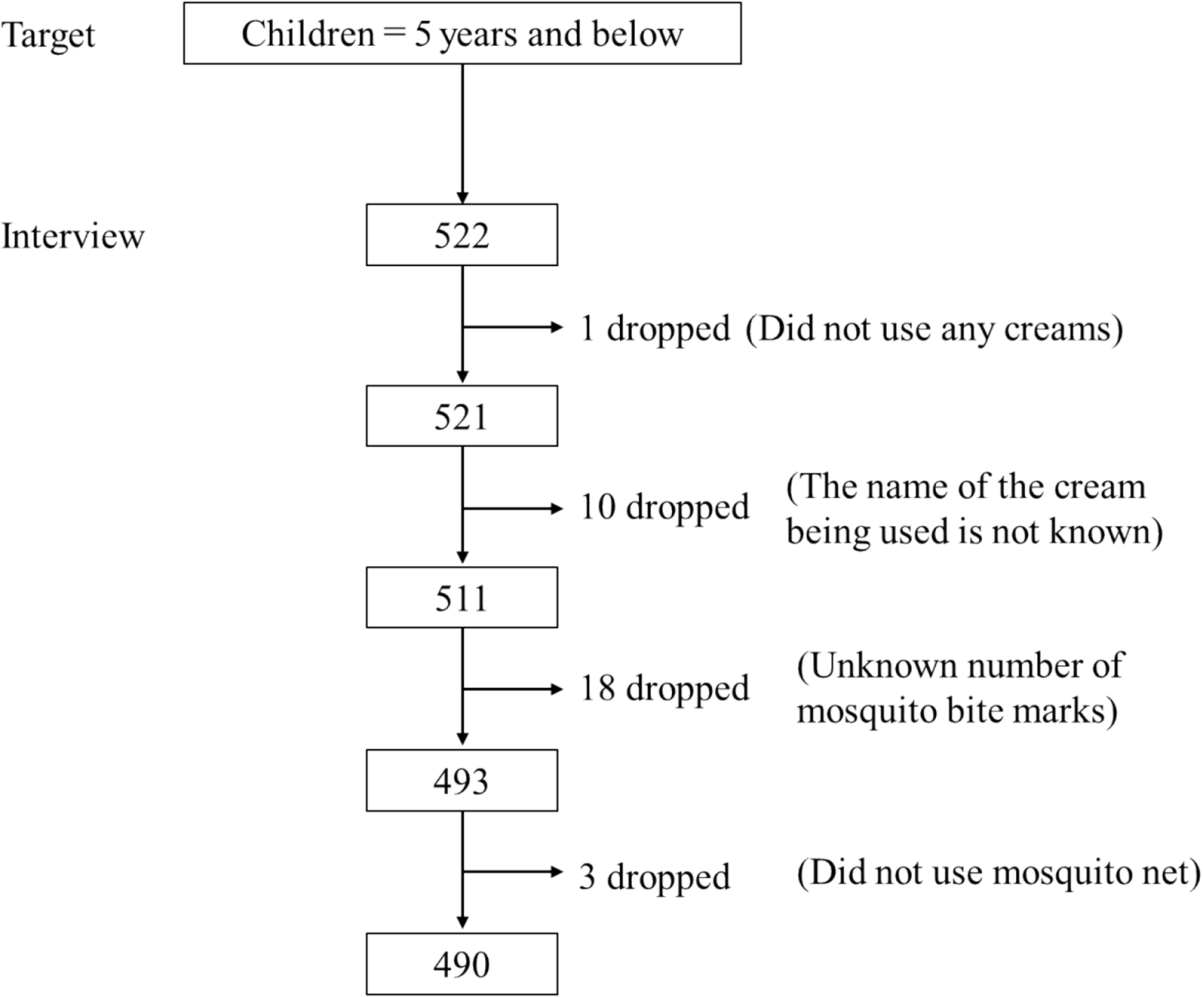
Flowchart of Inclusion Criteria in the final sample

Cream types used for each child were categorized into three groups: coconut oil, plant-based, and synthetic based on the active ingredients in the users’ daily moisturizers. The ‘synthetic’ group was composed of mainly petrolatum and the other creams (*n* = 383) whose ingredient’s had no known repellent effects. Since these creams had similar ingredients such as glycerin and had mainly moisturizing properties, they were grouped with petrolatum users into ‘synthetic’ (following Bouslimani et al. [41]). All users of coconut oil (*n* = 32) were selected from the rest as this was the target active ingredient to investigate for its relationship to mosquito bites. The plant-based category consisted of any herbal based or natural ingredient creams (*n* = 75). Data pre-processing (Fig. 4) involved systematically identifying and addressing missing values through imputation or exclusion based on predefined criteria. Inconsistent or incomplete entries were reviewed and corrected to ensure data integrity. The final data set was refined to maintain reliability and consistency for analysis. SES indicators, including parental education, income, and asset ownership, were analyzed using Principal Component Analysis (PCA) to create a composite SES index [42]. Behavioral variables were categorized for analysis and converted to dummy variables for analysis [40]. Each category was coded to binary as zeros and ones with the first category dropped off to avoid the dummy variable trap that can result in high multicollinearity [40]. Climate data (temperature and rainfall) were accessed from the ERA5 reanalysis data set [61] accessed from: https://cds.climate.copernicus.eu/datasets/derived-era5-single-levels-daily-statistics?tab=overview. The count and percentage of each variable was calculated by their categories, e.g., gender was separated into male and female across the three cream types (coconut oil, plant based and synthetic). For each variable a chi squared test [40] was performed to see if there was a statistical difference across the cream types per variable.

### Regression analysis

The outcome variable was the number of mosquito bite marks. Fixed effects included environmental (temperature, rainfall), behavioral, and demographic predictors. Categorical variables were encoded using dummy variables. Due to overdispersion in the mosquito bite count data (*α* = 4.66), Negative Binomial regression was employed for both simple and multiple regression analyses [44]. Variables for multiple regression were selected based on Cramer’s V matrix (see Appendix S2), with variables that had a strong correlation above 70% were removed, based on Pearson’s chi-square cutoff [40]. Although data were collected using random cluster sampling, villages were used as the random intercept instead of the original clusters, as they explained greater variance in the outcome [40]. This approach adjusted for within-village correlation and improved model fit by capturing variance at the village level. The model was fit using maximum likelihood estimation in Python's stats models.

## Results

### Blood feeding laboratory experiment

**Fig. 5:**
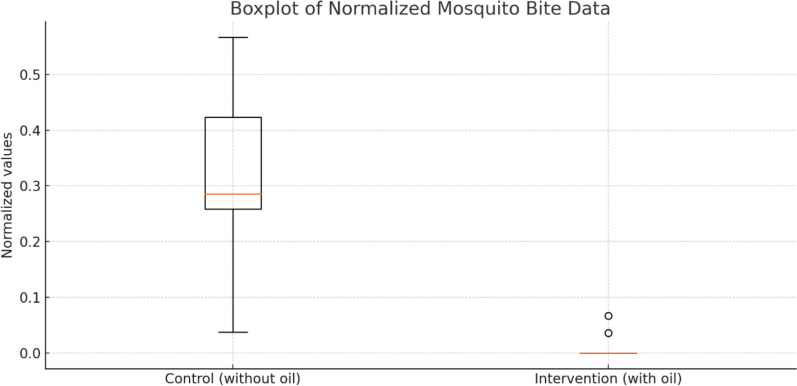
Normalized percentage of blood-feeding mosquitoes for both control and intervention (with coconut oil) groups over the number of experiments

The control group showed considerable variability in the normalized bite counts, with values ranging from approximately 0.04 to 0.57 (Fig. 5). The intervention group exhibited consistently low normalized bite counts, predominantly at zero with slight increases observed in only two trials (0.04 and 0.07) (Table 1). The control group exhibited an average blood-feeding rate of 31.0%. In contrast, the intervention group, treated with coconut oil, had a lower average blood-feeding rate of 1.14%. The difference between the control and intervention groups was statistically significant (*p* < 0.01). The Fishers exact test indicated that a majority of the experiments between use of coconut oil and control (excluding 7 and 8) displayed a statistically significant difference. To adjust for the repeated experiments and determine an overall effect, the pooled Mantel–Haenszel odds ratio was 0.06 which suggested that the odds of mosquitoes’ blood feeding for the coconut oil treated sides were lower than control sides. Furthermore, the Mantel–Haenszel chi-square statistic (79.82; *p* = 0.01) confirmed that the reduction of blood feeding in the coconut oil treated experiments was statistically significant.

**Table 1:** Result of odds ratio and Fisher’s exact test for each mosquito blood feeding experiments across coconut oil treated and control groups

Experiment no	OR	Fisher’s exact test (*p* value)
1	0.01	< 0.01
2	0.05	0.01
3	0.05	< 0.01
4	0.04	< 0.01
5	0.04	< 0.01
6	0.07	< 0.01
7	0.13	0.24
8	0.35	1.00
9	0.07	< 0.01

### Descriptive analysis of variables associated with cream type use

Coconut oil was predominantly used on younger children, with 62.5% of users being aged 0–1 years, compared to 33.3% for plant-based cream and 36.3% for synthetic cream (Table 2). A significant difference was observed between age intervals and the type of cream used (*p* = 0.04). Conversely, the age group 4–5 years had the smallest percentage of coconut oil users (9.4%) compared to around 25% for the other groups. Synthetic cream users were relatively evenly distributed across all age groups.

**Table 2:** Comparison of demographic and behavioral characteristics among participants using different types of creams (Coconut Oil, Plant Based and Synthetic) in Homa Bay County, Kenya

Variable	Coconut oil (*n* = 32)	Plant based (*n* = 75)	Synthetic (*n* = 383)	Chi-square	*p* value
Age				10.24	0.04*
0 to 1	20 (62.5%)	25 (33.3%)	139 (36.3%)		
2 to 3	9 (28.1%)	30 (40.0%)	141 (36.8%)		
4 to 5	3 (9.4%)	20 (26.7%)	103 (26.9%)		
Gender				0.53	0.77
Female	17 (53.1%)	36 (48.0%)	201 (52.5%)		
Male	15 (46.9%)	39 (52.0%)	182 (47.5%)		
SES				9.78	0.13
Low	11 (34.4%)	19 (25.3%)	90 (23.5%)		
Lower middle	9 (28.1%)	13 (17.3%)	106 (27.7%)		
Upper middle	4 (12.5%)	17 (22.7%)	99 (25.8%)		
High	8 (25.0%)	26 (34.7%)	88 (23.0%)		
Bath period				9.21	0.06
Morning (7:00–11:00)	2 (6.2%)	2 (2.7%)	14 (3.7%)		
Afternoon (12:00–16:00)	16 (50.0%)	57 (76.0%)	229 (59.8%)		
Evening (17:00–21:00)	14 (43.8%)	16 (21.3%)	140 (36.6%)		
Bath frequency				13.08	0.01*
Low (one time)	2 (6.2%)	2 (2.7%)	14 (3.7%)		
Medium (two times)	16 (50.0%)	57 (76.0%)	229 (59.8%)		
High (three/more times)	14 (43.8%)	16 (21.3%)	140 (36.6%)		
Cream application period				6.89	0.14
Morning (7:00–11:00)	3 (9.4%)	2 (2.7%)	22 (5.7%)		
Afternoon (12:00–16:00)	18 (56.2%)	57 (76.0%)	237 (61.9%)		
Evening (17:00–21:00)	11 (34.4%)	16 (21.3%)	124 (32.4%)		
Cream application frequency				9.69	0.05*
Low (one time)	13 (40.6%)	44 (58.7%)	184 (48.0%)		
Medium (two times)	13 (40.6%)	28 (37.3%)	172 (44.9%)		
High (three/more times)	6 (18.8%)	3 (3.9%)	27 (7.0%)		
Net entry time				6.27	0.18
Early (17:00–18:00)	3 (9.4%)	2 (2.7%)	14 (3.7%)		
Normal (19:00–20:00)	12 (37.5%)	39 (52.0%)	155 (40.5%)		
Late (21:00–22:00)	17 (53.1%)	34 (45.3%)	214 (55.9%)		
Net entry after cream application				1.24	0.54
Under 5 h	21 (65.6%)	41 (53.9%)	214 (55.6%)		
Over 5 h	11 (34.4%)	34 (45.3%)	169 (44.1%)		
Clothing (top)				1.42	0.49
Long sleeve	28 (87.5%)	62 (82.7%)	336 (87.7%)		
No/short sleeve	4 (12.5%)	13 (17.3%)	47 (12.3%)		
Clothing (bottom)				3.77	0.15
Long pants	28 (87.5%)	60 (80.0%)	338 (88.3%)		
No/short pants	4 (12.5%)	15 (20.0%)	45 (11.7%)		
Prevention methods (excl. mosquito net)				1.92	0.38
Never used	26 (81.2%)	57 (76.0%)	317 (82.8%)		
Used	6 (18.8%)	18 (24.0%)	66 (17.2%)		
Eaves				6.10	0.05*
Closed	8 (25.0%)	9 (12.0%)	40 (10.4%)		
Open	24 (75.0%)	66 (88.0%)	343 (89.6%)		
Region				14.76	0.02*
Gembe	14 (43.8%)	12 (16.0%)	88 (23.0%)		
Rangwa	10 (31.2%)	23 (30.7%)	125 (32.6%)		
Nyamrisra	1 (3.1%)	22 (29.3%)	83 (21.7%)		
Ngeri	7 (21.9%)	18 (24.0%)	87 (22.7%)		
Total	32	75	383		

The observed frequencies (and percentage in brackets) of people across each variable and with its specific categories are reflected for each cream type. The chi-squared test of independence was performed for each variable to see if there was significant association between that variable and the cream types. The larger the chi-squared value, the less chance of a relationship existing between the three cream types across that variable. The *p* value indicates the statistical significance of the chi-squared score

The bath period showed a near-significant association with cream type (*p* = 0.06), with coconut oil users predominantly bathing in the evening (43.8%), while plant-based cream users primarily bathed in the afternoon (76.0%). Bath frequency was significantly associated with the type of cream used (*p* = 0.01), with coconut oil users bathing more frequently compared to users of plant-based or synthetic creams. Although the cream application period did not exhibit a significant association (*p* = 0.14), the frequency of cream application was significantly associated with cream type (*p* = 0.05). Coconut oil users applied cream more frequently, indicating more consistent use of coconut oil.

Net entry time and the time between cream application and net entry did not show significant associations with cream type (*p* = 0.18 and *p* = 0.54, respectively). Clothing type, both top and bottom, also did not show significant associations with cream type (*p* = 0.49 for top and *p* = 0.15 for bottom). Similarly, the use of additional prevention methods was not significantly associated with cream type (*p* = 0.38). However, eaves status was significantly associated with cream type (*p* = 0.05). Coconut oil users were more likely to have closed eaves (25.0%) compared to users of plant-based (12.0%) and synthetic creams (10.4%), suggesting a potential interaction between environmental factors and cream choice.

Regional distribution showed a significant association with cream type (*p* = 0.02). In Gembe, a higher proportion of children used coconut oil (43.8%) compared to plant-based (16.0%) and synthetic creams (23.0%). In Nyamrisra, plant-based cream was more commonly used (29.3%), while Rangwa exhibited a balanced distribution across all cream types.

Overall, the results indicate that coconut oil is more commonly used by younger children and those who bathe and apply cream more frequently. Gender and socioeconomic status (SES) did not significantly influence cream choice. However, behavioral patterns, such as bath and cream application frequencies, as well as regional and environmental factors like eaves status, were significantly associated with the type of cream used. While these findings do not establish a direct link between coconut oil and mosquito bite count incidence, they highlight significant differences between coconut oil users and other cream users in terms of demographic, environmental, and behavioral factors.

### Regression model

**Fig. 6:**
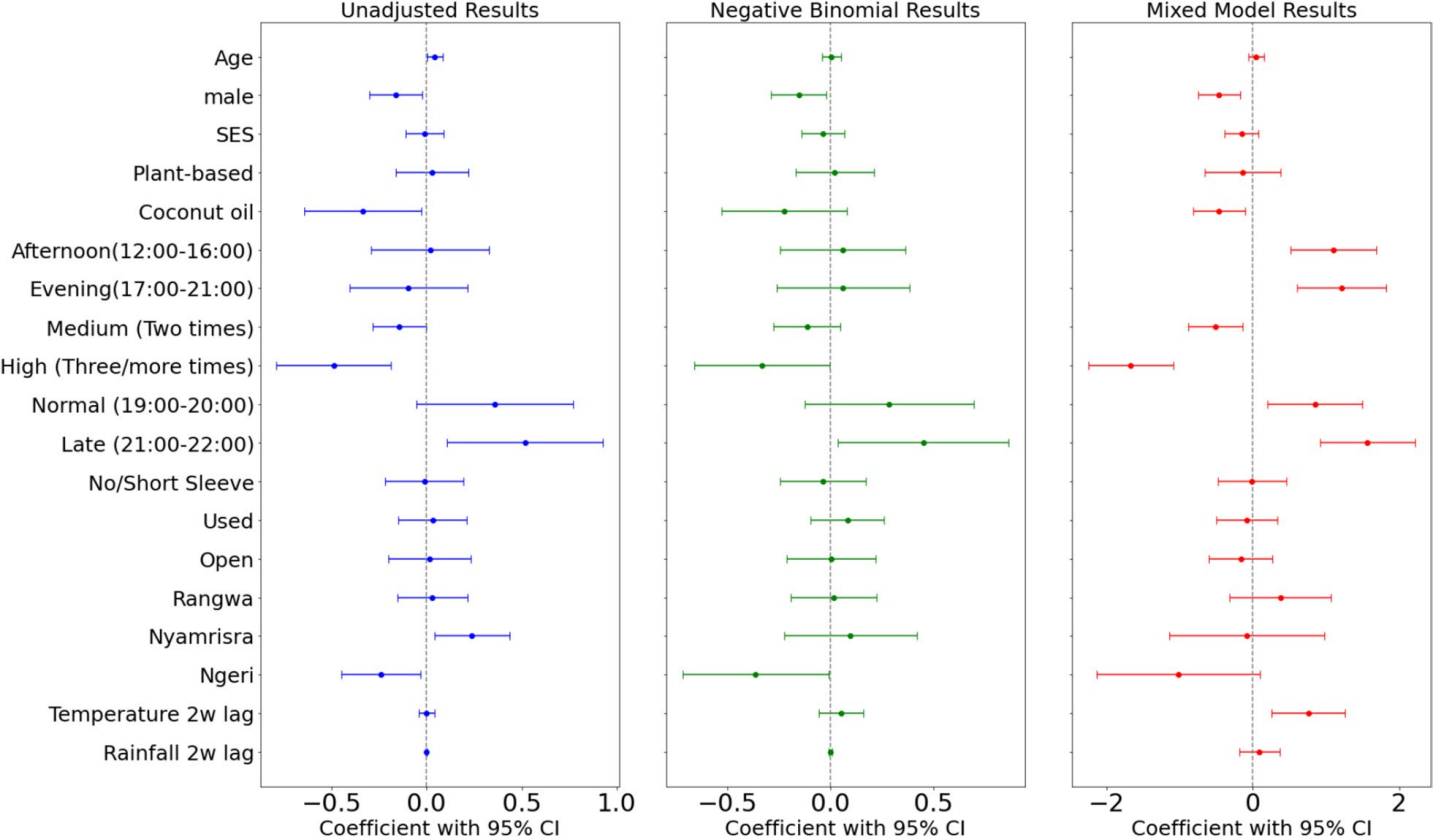
Caterpillar plots for predictors coefficients (with 95% confidence interval) derived from (**a**) simple regression, (**b**) negative binomial multiple regression, and (**c**) mixed linear model using bite marks as independent variable. The *x*-axis represents the coefficient values, where points indicate the estimated effect size, and horizontal lines represent the 95% confidence intervals. The dashed vertical line at 0 represents no effect. Coefficients whose confidence intervals do not cross zero are considered statistically significant

Three models were applied to determine associations between the predictor variables of the study and the bite mark count outcome (Table 3; Fig. 6). The Unadjusted model shows crude associations, the Negative Binomial model adjusts for overdispersion, and the Mixed Effects model accounts for data clustering and random effects. The impact of various factors on the number of bite marks was analyzed using negative binomial simple regression with each variable’s relation with mosquito bite marks, revealing notable differences in their significance and effect sizes. With synthetic creams as a reference, using coconut oil showed a statistically significant difference, reducing mosquito bite counts by 28.6% (RR = 0.71; 95%CI − 0.64 to − 0.03). Late net entry time also had a significantly increasing effect on mosquito bite counts by 68.0% (RR = 1.68; 95%CI 0.11 to 0.93) and normal net entry time was also associated with a 43.2% increase in bite counts compared to early net entry indicating net entry had a protective effect if done early.

**Table 3:** Co-efficient (Coef.), *p* values (*p*-val), and percentage effect size (ES%) for various factors influencing mosquito bite marks across three models: unadjusted, adjusted negative binomial (NB), and mixed model

	Unadjusted			Adjusted (NB)				Mixed model		
	Coef.	*p*-val	ES (%)		Coef.	*p*-val	ES (%)	Coef.	*p*-val	ES (%)
Age	0.04	0.04*	4.50		0.01	0.81	0.55	0.04	0.42	1.50
Gender (female)										
Male	− 0.16	0.02*	− 14.89		− 0.15	0.03*	− 14.25	− 0.46	< 0.01*	− 15.74
SES	− 0.01	0.84			− 0.03	0.52	− 3.37	− 0.16	0.20	− 5.28
Cream types (synthetic)										
Plant-based	0.03	0.76	3.03		0.02	0.82	2.23	− 0.14	0.61	− 4.60
Coconut oil	− 0.34	0.03*	− 28.61		− 0.22	0.15	− 20.09	− 0.47	0.01*	− 15.84
Bath period (morning)										
Afternoon	− 0.01	0.96	− 0.93		–	–				
Evening	− 0.07	0.71	− 6.90		–					
Bath frequency (low/one time)										
Medium (two times)	− 0.04	0.63	− 3.52							
High (three/more times)	− 0.39	< 0.01*	− 32.47							
Cream app. period (morning)										
Afternoon	0.02	0.90	1.90		0.06	0.70	6.24	1.11	< 0.01*	37.65
Evening	− 0.09	0.55	− 9.06		0.06	0.70	6.41	1.22	< 0.01*	41.47
Cream app. frequency (low/one time)										
Medium (two times)	− 0.14	0.05*	− 13.31		− 0.11	0.17	− 10.73	− 0.51	0.01*	− 17.45
High (three/more times)	− 0.49	< 0.01*	− 38.59		− 0.33	0.05*	− 28.27	− 1.67	< 0.01*	− 56.87
Net entry time (early/17:00–18:00)										
Normal (19:00–20:00)	0.36	0.09	43.22		0.29	0.17	33.16	0.85	0.01*	28.96
Late (21:00–22:00)	0.52	0.01*	67.89		0.45	0.03*	57.27	1.57	< 0.01*	53.50
Clothing (top: long)										
No/short sleeve	− 0.01	0.92	− 1.11		− 0.04	0.74	− 3.47	− 0.01	0.96	− 0.44
Clothing (bottom: long)										
No/short pants	0.03	0.76	3.19							
Prevention methods (never used)										
Used	0.03	0.73	3.21		0.08	0.35	8.79	− 0.08	0.70	− 2.79
Eaves (closed)										
Open	0.02	0.89	1.56		0.00	0.97	0.43	− 0.17	0.46	− 5.66
Region (gembe)										
Rangwa	0.03	0.74	3.17		0.02	0.88	1.63	0.38	0.29	12.78
Nyamrisra	0.24	0.02*	26.89		0.10	0.55	10.34	− 0.09	0.87	− 2.93
Ngeri	− 0.24	0.03*	− 21.18		− 0.36	0.05*	− 30.41	− 1.02	0.07	− 34.72
Temperature 2w lag	0.00	0.98	0.05		0.05	0.33	5.48	0.76	< 0.01*	25.86
Rainfall 2w lag	0.00	0.84	− 0.05		0.00	0.73	0.13	0.09	0.52	3.07

Related to this, medium (RR = 0.87; 95%CI − 0.29 to 0.00) and higher cream use (RR = 0.61; 95%CI − 0.79 to − 0.19) each had a significant influence over the bite count with an increase in cream applications associated with a 13.0 and 38.0% decrease in mosquito bite counts, respectively. Related to cream application activity, evening cream application (RR = 0.91; 95%CI − 0.40 to 0.21) displayed patterns of reducing bite counts. High bath frequency was another notable factor, significantly reducing bite counts by 32.0% (RR = 0.68; 95%CI − 0.64 to − 0.15). While being male was associated with a 14.9% reduction in bite counts (RR = 0.85; 95%CI − 0.30 to − 0.02). Age indicated a statistically significant positive effect size (RR = 1.04; 95% CI 1.002 to 1.090), implying older children are at a 4.5% higher risk for increased mosquito bites per unit increase in age. Regional differences were also observed. Compared to Gembe, Nyamrisra exhibited a more notable 26.9% increase (RR = 1.27; 95%CI 0.04 to 0.44). Conversely, Ngeri was associated with a 21.2% reduction in bite counts (RR = 0.79; 95%CI − 0.45 to − 0.03). The region, gender and age may also have an effect on the risks related to mosquito bites.

The multiple regression analysis using a negative binomial model identified significant effects of several covariates on mosquito bite counts. Being male was associated with a 14.3% reduction in bite counts (RR = 0.86; 95%CI − 0.29 to − 0.02; *p* = 0.03). High cream application frequency significantly reduced bites by 28.3% (RR = 0.72; 95%CI − 0.66 to − 0.01; *p* = 0.04). Entering the mosquito net late (more than 5 h after cream application) was linked to a 57.3% increase in bite counts (RR = 1.57; 95%CI 0.04 to 0.87; *p* = 0.03). Regional differences also emerged, with one area showing a significant 30.4% reduction in bites (RR = 0.70; 95%CI − 0.72 to − 0.01; *p* = 0.05). While coconut oil showed a reduction in bite counts by 20.6% (RR = 0.79; 95%CI − 0.54 to 0.07), the results were not statistically significant (*p* = 0.13). These results emphasize the protective effects of cream application frequency, male gender, and specific regional characteristics, as well as the critical role of timing in mosquito net usage.

**Fig. 7:**
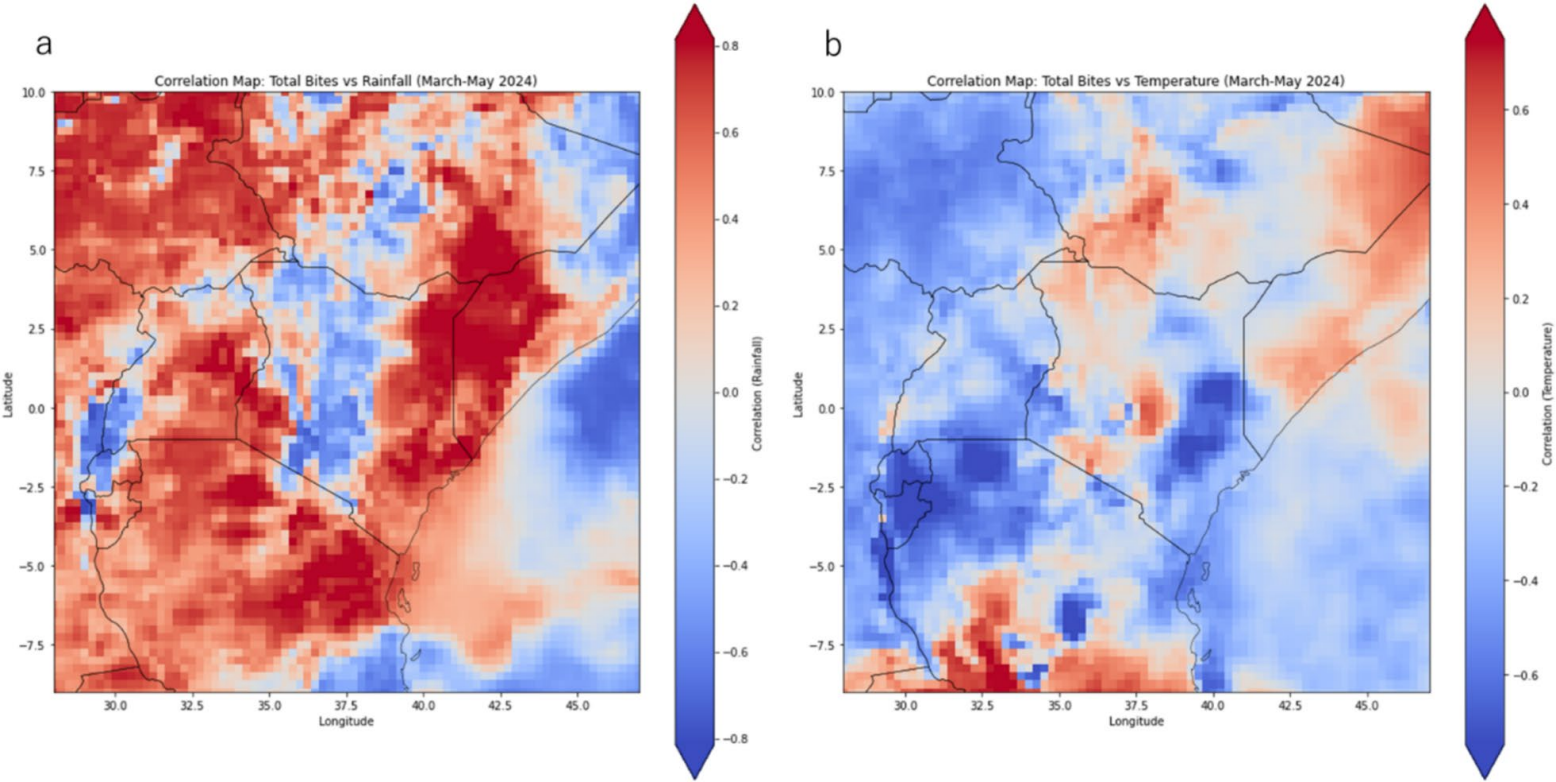
Correlation maps between total bite counts and rainfall (left) and temperature (right). Correlation is between total bite counts from March to May 2024 and the corresponding mean temperature and total rainfall. Red regions indicate a positive correlation between temperature/rainfall and bite counts, while blue indicates negative correlation

The mixed-effects model (Table 3, Fig. 7), including village as a random effect, revealed several key associations with mosquito bite counts. Temperature lagged by 14 days significantly increased bite counts by 25.9% (RR = 2.14; 95%CI 0.26 to 1.26; *p* = 0.003). Being male was associated with a 15.7% reduction in bites (RR = 0.63; 95%CI − 0.75 to − 0.17; *p* = 0.002). High cream application frequency significantly reduced bites by 56.9% (RR = 0.19; 95%CI − 2.25 to − 1.09; *p* < 0.001), while medium cream application reduced bites by 17.5% (RR = 0.60; 95%CI − 0.89 to − 0.14; *p* = 0.007). Late cream application times were associated with a 41.5% increase in bites (RR = 3.38; 95%CI 0.61 to 1.83; *p* < 0.001). Entering the mosquito net late significantly increased bite counts by 53.5% (RR = 4.81; 95%CI 0.93 to 2.22; *p* < 0.001). Coconut oil users experienced a 15.8% reduction in bites (RR = 0.63; 95%CI − 0.82 to − 0.11; *p* = 0.01), though synthetic creams did not show significant results. These findings emphasize the protective effects of frequent cream application, gender, and net entry timing, alongside temperature's influence. The group variance (17.0%) reflects variability across villages. By using village groups as the random effect, the model is able to correctly account for climate influences and cream type effects, indicating that there is significant variability between villages. This suggests that village-level factors, such as environmental conditions, local mosquito populations, housing structure, and behavioral patterns, play an important role in influencing mosquito bite counts. The inclusion of the random effect helps account for these contextual differences, improving the precision of the estimated effects of climate and cream types while recognizing that villages are not homogeneous in their exposure or response to these factors.

### Climate correlation maps

Climate and bite count correlation maps summarizing the bites data from March to May were generated to determine the regions of temperature and rainfall that have strong relationships with the bite count patterns (Fig. 7a). Positive correlations in central and eastern regions of Kenya suggest that higher rainfall levels are associated with an increase in mosquito bites. For temperature (Fig. 7b), the negative correlations across most regions indicates that higher temperatures are linked to a reduction in mosquito bites. However just south of Kenya and over the ocean to the countries coastline the temperature has high correlation to bite counts. When a global extent was used for the correlation was used, there were high positive correlations over the South Pacific and Arabian sea locations (Appendix S5).

## Discussion

### Coconut oil’s effectiveness as a repellent

This study showed that coconut oil users experienced a statistically significant reduction in mosquito bite counts compared to users of synthetic creams (Table 3). Specifically, coconut oil was associated with a 15.8% reduction in bites. In this study’s laboratory experiments, coconut oil-treated membranes resulted in only 1% blood-feeding rates for *Anopheles* mosquitoes compared to 31% in the control group, indicating a strong repellent effect. These results align with previous findings, such as Zhu et al*.* [13], which reported over 93% protection against *Aedes* mosquitoes using derived fatty acids from coconut oil. The strong repellence observed in these studies underscores the potential of coconut oil as a viable natural alternative to chemical repellents such as DEET or other established natural repellents such as citronella oil [5].

Field studies on the repellent effects of pure coconut oil remain scarce. Kebede et al*.* [45] demonstrated its efficacy against sandflies, providing 81–83% protection in laboratory settings and 86–94% in field conditions. Given that sandflies and mosquitoes share similar blood-feeding behaviors [46], these results lend credibility and are built upon by the observed repellence in the current study. Furthermore, the significant reduction in mosquito bites among coconut oil users in our study complements the findings of Soonwera and Phasomkusolsil [47], who reported 98% protection when coconut oil was used as a base for Ylang ylang and lemongrass oils.

Our findings also suggest that coconut oil’s repellent properties persist even when applied in its pure form. This is particularly relevant in regions where conventional repellents are either unavailable or unaffordable. In addition, the reduction in mosquito bite counts highlights coconut oil’s potential for public health interventions, particularly in malaria-endemic areas like Homa Bay County, Kenya where coconut oil products are cheap and abundant even in the rural and poor regions.

### Frequency and timing of cream application

The frequency of cream application emerged as an important factor influencing mosquito bite counts. High-frequency users experienced a significant 57% reduction in bites (Table 3), while medium-frequency users saw a 17% reduction. These findings align with Hazerika et al*.* [48], who emphasized that the efficacy of topical repellents depends on application frequency due to their low volatility. This mechanism may explain the effect observed in this study. Even though a majority of synthetic cream users also applied the cream up to two times a day (Table 2), compared to coconut oil users there was no repellent effect (Fig. 6). This infers that the more times coconut oil is applied through the day the more mosquito bite incidence can be decreased. Well known plant-based repellents such as Citronella usually have a repellency of over 6 h against Anopheles mosquitoes [5], while previous studies indicated coconut oil mixed with Vaseline (petroleum jelly) displayed 100% repellency for 45 min, but this was increased up to 127 min by increasing coconut oil concentration from 25% [5, 24].

The timing of application also played a significant role (Table 3). Most coconut oil users in the study applied the cream in the afternoon or evening, incidentally aligning with the peak biting periods of Anopheles and Culex mosquitoes [49, 50]. Late cream application, however, was associated with a 41% increase in bite counts, suggesting that timely application is essential for optimal protection.

The findings related to cream application reinforce the importance of behavior modification in mosquito bite prevention. Encouraging regular and timely application of coconut oil or other repellents could enhance their protective effects, particularly in regions where conventional repellents are scarce or expensive. Furthermore, integrating coconut oil into existing skincare routines—such as mixing it with petroleum jelly—could increase its adoption and effectiveness [24, 47].

### Gender, age and vulnerability

The study also found that being male was associated with a 15% reduction in mosquito bites. This finding could be attributed to differences in skin temperature, odor profiles, and activity patterns between genders, which influence mosquito attraction [51, 64]. Higher skin temperatures in males may result in a different composition of volatile organic compounds (VOCs), which can act as attractants or repellents to mosquitoes [23, 51]. In addition, males may have differing activity levels that influence exposure to mosquitoes, such as spending less time outdoors during peak ting hours [65]. Regardless of gender, younger children would typically spend more time indoors during late hours, which would decrease exposure to nocturnal mosquitoes [73]. This study has in fact shown that older children have a 4.5% higher risk for mosquito bites compared to younger ages (Table 3). The difference in time spent outside and care from parents could be potential factors influencing bite counts among different ages; however, household structures and use of bed nets have still been reported to influence bite outcomes [73]. Other possibilities for the gender disparity should be explored, as the existing literature indeed reports individual variability in mosquito host-seeking behavior based on unique biochemical profiles [51]. Future studies should explore how physiological and behavioral differences between genders influence mosquito attraction to tailor targeted interventions and address possible at-risk groups based on their sex for instance. If the main cause of this gender disparity is indeed linked to biogeochemical profiles, the application of repellents may help alter them enough to prevent and decrease attraction to mosquitoes [8, 66].

### Environmental and behavioral factors

The inclusion of village as a random effect revealed a group variance of 17%, highlighting the importance of contextual factors that data may not have been available for, including but not limited to local mosquito populations, housing structures, and extraneous behavioral or environmental patterns [33].

Related to the environmental effects, temperature lagged by 14 days was associated with a 26% (Table 3) increase in bite counts, emphasizing the role of climatic conditions in mosquito activity. Temperature directly impacts mosquito physiology, including their metabolic rates and reproduction cycles [42]. Studies have shown that higher temperatures accelerate the development of mosquito larvae and shorten the gonotrophic cycle, thereby increasing mosquito populations [52]. The findings from this study supports the assumption that warmer temperatures are related to increased mosquito abundance, which lead to more bite incidence. Furthermore, warmer temperatures enhance the flight activity and host-seeking behavior of mosquitoes, leading to increased biting rates [53], which are most pronounced when temperatures remain within an optimal range (25–28 °C) for mosquito activity [64, 67]. The negative correlations between the temperature across most of Kenya and bite count (Fig. 7) is potentially due to temperatures exceeding the optimal range for mosquito activity [52, 53], reducing the abundance and bites as a result. In Homa Bay, Kenya, the warm climate likely creates ideal conditions for mosquito proliferation and heightened bite risk, further substantiating the findings of temperature and bite mark associations of this study.

Rainfall patterns also play a critical role in mosquito dynamics [42]. While this study did not directly measure rainfall, its influence is inferred through its role in creating breeding habitats. Rainfall provides stagnant water, which serves as breeding sites for *Anopheles* and *Culex* mosquitoes. However, excessive rainfall can flush out these habitats, temporarily reducing mosquito populations [49, 54]. In regions like Homa Bay, where rainfall patterns are seasonal, the interplay between rainfall and temperature creates cyclical peaks in mosquito abundance, directly influencing bite incidence. In fact, as seen in this study (Fig. 6), while the regression analysis did not detect rainfall at 2 weeks lag to be significant to bite counts, the spatial correlations (Fig. 7) indicate there are indeed regions of the country where the rainfall activity is highly correlated with bite outcomes. Similar findings by Martineau et al*.* [55] and Behera et al*.* [56] indicate that these spatial correlations with climate and inferred mosquito activity are useful for indicating how climate variables over different regions influence health outcomes, such as malaria. The spatial correlations found here, therefore, could be used in future investigations to understand the spatial patterns between mosquito activity, vector-borne diseases and climate in west Kenya.

Late net entry (21:00–22:00) was another significant factor, associated with a 54% increase in bite counts (Table 3). This aligns with the nocturnal biting patterns of *Anopheles* mosquitoes and underscores the importance of early net use in reducing exposure [49]. Behavioral interventions related to net entry time, such as encouraging earlier net entry, could significantly reduce mosquito bite risk and, consequently, malaria transmission.

### Implications for malaria prevention

The literature on pure coconut oil as a repellent is scarce with most studies using derived acids and products from coconut [5, 13, 25]. This study adds to the literature and demonstrates that pure coconut oil does in fact have repellent properties and can offer protection from mosquito bites. Homa Bay County has one of the highest malaria prevalence rates in Kenya, driven by the abundance of *Anopheles* mosquitoes [27, 28]. While the Kenyan government implements malaria prevention strategies in regions like Homa Bay, by providing free mosquito nets every 4 years, free treatment for children under five, and free prophylactic medication for pregnant women [68]. However, obtaining these nets is complicated due to inadequate information systems and distribution infrastructure [57]. It is also difficult to access local clinics, which often lack sufficient medications [70]. Therefore, while mosquito nets are effective [69] and anti-malaria drugs can decrease risk, personal preventive measures are also crucial to provide protection to vulnerable, poor and remote regions. In this context, coconut oil offers a cost-effective and sustainable alternative for mosquito bite prevention and by extension malaria mitigation. Compared to chemical repellents, which can cause skin irritation and are often expensive [58], coconut oil is natural, affordable, and widely available in rural Kenya. Its additional benefits for skin health—including anti-inflammatory and skin-protective properties [59]—further enhance its appeal. Raising awareness about its repellent properties and promoting its integration into daily routines could significantly reduce mosquito bite incidence in vulnerable populations and also decrease overall risk from mosquito borne diseases, such as malaria and the resultant health complications.

### Limitations and future directions

The present study used a cross-sectional design, which limits the ability to determine the temporal relationship between mosquito bites and cream application. Since data on bite occurrence and cream application were collected retrospectively, it is unclear whether the cream was applied before or after the bites, leading to potential misclassification bias. Therefore, the field results for coconut oil effectiveness should be discerned with caution. The reliance on self-reported data for various variables may have introduce recall bias. Participants may have inaccurately remembered or reported their behavior, especially given that mosquito bites often go unnoticed at the time of occurrence. For instance, this could lead to misestimation of the association between cream application and bite counts. Future studies could minimize this bias by incorporating real-time logging methods, such as diary records or mobile applications, to improve accuracy. There was also no established method for identifying mosquito bite marks; therefore, we had to make the definition and get training to consistently identify mosquito bite marks. However, variability in individual skin reactions and observer subjectivity may have influenced bite count accuracy. This issue is particularly relevant because some mosquito bites may not elicit visible skin reactions, leading to potential underestimation of bite counts, especially among individuals with lower skin sensitivity or rapid inflammatory resolution. Future studies could explore the use of imaging diagnostics to enhance the accuracy and scalability of such observations. Furthermore, experimental studies under controlled conditions are needed to validate our findings and confirm whether the observed associations between cream use and mosquito bites hold under standardized environmental and behavioral conditions. Further research should also investigate the long-term effects of coconut oil application, its efficacy against different mosquito species, and its integration with other preventive measures. Exploring community-based interventions to promote its use could enhance its impact in malaria-endemic regions.

## Conclusion

This study investigated the potential efficacy of coconut oil as a repellent for decreasing mosquito bite counts. Coconut oil demonstrated possible effectiveness in decreasing blood-feeding behavior for mosquitoes in laboratory settings and also indicated some association with reduced mosquito bites in the field study. These findings add to the literature on coconut oils potential as a natural mosquito repellent, while also underscoring the complex nature of mosquito bite risk and associated malaria risk among young children in malaria-endemic regions. The findings emphasize the potential of integrated, behavior-based interventions, such as frequent cream application during peak biting times of *Anopheles* mosquitoes and earlier mosquito net entry, to mitigate mosquito bite risk. Further investigation into the effectiveness of these protective behaviors can strengthen public health knowledge to reduce mosquito bites and associated malaria burdens in Homa Bay County, Kenya, and similar regions. To better understand the specific impact and duration of protection offered by pure coconut oil, rigorous laboratory studies are recommended to explore its underlying mechanisms. In addition, future field studies with larger sample sizes and controlled designs are necessary to conclusively establish its efficacy as a mosquito repellent.

## Supplementary Information

Supplementary Material 1.

## Data Availability

No data sets were generated or analysed during the current study.
