# Supplementary material for: Potential of coconut oil as a mosquito repellent
Source: Trop Med Health. 2025 Apr 23;53:57. doi: 10.1186/s41182-025-00714-8 (PMC12016410; doi:10.1186/s41182-025-00714-8)
Supplement: Supplementary file 1 — Supplementary Material 1. [file 41182_2025_714_MOESM1_ESM.docx]

**Appendices**

**Appendix 1.**Table of questions used in field questionnaire

| **Question ID** | **Questions** |
| --- | --- |
| i | Date of interview |
| ii | Current location |
| iii | Name of parent/guardian |
| iv | New member's name |
| v | Date of birth (New member) |
| ii | Gender(New member) |
| iii | Can interview be conducted? |
| iv | Respondent's relationship to the child |
| Cream Application Behavior | |
| 1 | Did you apply any creams/oils to the child's skin yesterday? |
| 2a | Why are you applying any creams/oils on the child? |
| 2b | If other, please fill in details |
| 3 | Who or what influences applying any creams/oils on the child? |
| 4 | Have you changed the cream/oil in the past week? |
| 5 | How many times did you apply the cream/oil yesterday? |
| 6a | When did you apply the cream/oil? (1st time) |
| 6b | If other, please fill in details |
| 6c | What time did you apply the cream/oil? (1st time) |
| 6d | When did you apply the cream/oil? (2nd time) |
| 6e | If other, please fill in details |
| 6f | What time did you apply the cream/oil? (2nd time) |
| 6g | When did you apply the cream/oil? (3rd time) |
| 6h | If other, please fill in details |
| 6i | What time did you apply the cream/oil? (3rd time) |
| 7a | Which part did you apply the cream/oil on the child yesterday? |
| 7b | Which part did you apply the cream/oil on the child specifically? |
| Bathing Behavior | |
| 8 | How many times did the child take a bath yesterday? |
| 9a | What time did the child take a bath yesterday? (1st time) |
| 9b | What time did the child take a bath yesterday? (2nd time) |
| 9c | What time did the child take a bath yesterday? (3rd time) |
| Mosquito Net and Clothing Behavior | |
| 10 | What time did the child enter the mosquito net last night? |
| 11 | What top was the child wearing when sleeping last night? |
| 12 | What bottom was the child wearing when sleeping last night? |
| 13a | Why did the child wear long top/bottom last night? |
| 13b | If other, please fill in details |
| 14 | What time did the child get out of mosquito net this morning? |
| Malaria and Prevention | |
| 15 | Has the child ever been diagnosed for Malaria in a clinic / hospital in the past 3 months? |
| 16 | How many times did the child have Malaria in the past 3 months? |
| 17 | Apart from mosquito net, do you usually use any other methods to prevent the child from mosquito bites? |
| 18a | Which methods do you usually use? |
| 18b | If other, please fill in details |
| 19 | Are you aware of any cream that prevents mosquito bites? |
| 20 | Have you ever applied mosquito repellent cream for the child? |
| 21 | If there was a cream that could reduce number of mosquito bites by 50%, would you apply it on the child? |
| 22 | If that cream was 50ksh/50g, would you want to buy it? |
| Mosquito Bite and Cream Type Questions (Ask last) | |
| 23 | Did the child get mosquito bite yesterday? |
| 24 | [Our side] How many mosquito bite's marks does the child have? |
| 25a | What kind of creams did you apply on the child? (Number of cream) |
| 25b | If other, please fill in details |
| 26a | What is the reason for choosing that cream or oil? |
| 26b | If other, please fill in details |
| 27 | If you change the cream/oil, what kind of creams/oils did you change from? (Number of cream) |
| 28 | Who or what influenced the choice of that cream? |
| 29 | Please write down any comments |
| 30 | Ask to take picture of mosquito bites |
| SES Questions | |
| 31a | Material of the wall |
| 31b | Material of the roof |
| 31c | Material of the floor |
| 32 | Do you stay in your own house? |
| 33a | How many rooms does this structure have? |
| 33b | Does this structure have (an) eave(s)? |
| 34a | Has this structure ever got the Indoor Residual Spraying done? |
| 34b | As to the most recent spraying, who did the spraying? |
| 34c | Month of the most recent spraying |
| 34d | Year of the most recent spraying |
| 35 | Highest grade of education completed on child's mother |
| 36a | Primary occupation on Child's mother |
| 36b | How many days did child's mother work in the last four weeks? |
| 36c | Highest grade of education completed on child's father |
| 37a | Primary occupation on Child's father |
| 37b | How many days did child's father work in the last four weeks? |
| 37c | How much income did members of your household make in the last four weeks? |
| 38 | How much was it take for school fee last year?(Ksh) |
| 39 | How much was it take for treatment last 4 weeks?(Ksh) |
| 40 | How much did child's mother and father use for airtime last four weeks? |
| 41 | Do members of your household have any insurance? |
| 42a | What is the main source of drinking water for members of your household |
| 42b | Where is that water source located? |
| 43c | How long does it take to go there, get water, and come back in minutes? |
| 44 | What kind of toilet facility do members of your household usually use? |
| 45 | What type of fuel does your household mainly use for cooking? |
| 46a | Does this household have access to electrical grid? |
| 46b | How much did you pay for electricity in the last month?(Ksh) |
| 46c | Does this household have solar panels? |
| 47 | What is the area size of land under cultivation (acres) in this season? |
| 48a | Does this household own any livestock, herd, other farm animals, or poultry? |
| 48b | How many of this type of COWS does this household own? |
| 48c | How many of this type of BULLS does this household own? |
| 48d | How many of this type of YOUNG_BULLS does this household own? |
| 48e | How many of this type of HEIFERS does this household own? |
| 48f | How many of this type of CALVES does this household own? |
| 48g | How many of this type of GOATS does this household own? |
| 48h | How many of this type of SHEEPS does this household own? |
| 48i | How many of this type of PIGS does this household own? |
| 48j | How many of this type of DONKEYS does this household own? |
| 48k | How many of this type of CHICKENS does this household own? |
| 49a | Does any member of this household own following types of assets? |
| 49b | How many of this type of asset does this household own? (Radio) |
| 49c | How many of this type of asset does this household own? (Television) |
| 49d | How many of this type of asset does this household own? (DVD player, Recoder) |
| 49e | How many of this type of asset does this household own? (PC) |
| 49f | How many of this type of asset does this household own? (Mobile phone) |
| 49g | How many of this type of asset does this household own? (Refrigerator) |
| 49h | How many of this type of asset does this household own? (Bicycle) |
| 49i | How many of this type of asset does this household own? (Motorcycle, Moterscooter) |
| 49j | How many of this type of asset does this household own? (Animal drawn cart) |
| 49k | How many of this type of asset does this household own? (Vehicle, Truck) |
| 49l | How many of this type of asset does this household own? (Motorboat) |
| 49m | How many of this type of asset does this household own? (Car) |
| 49n | How many of this type of asset does this household own? (Boat without motor) |

**Appendix 2**. Cramer’s V matrix


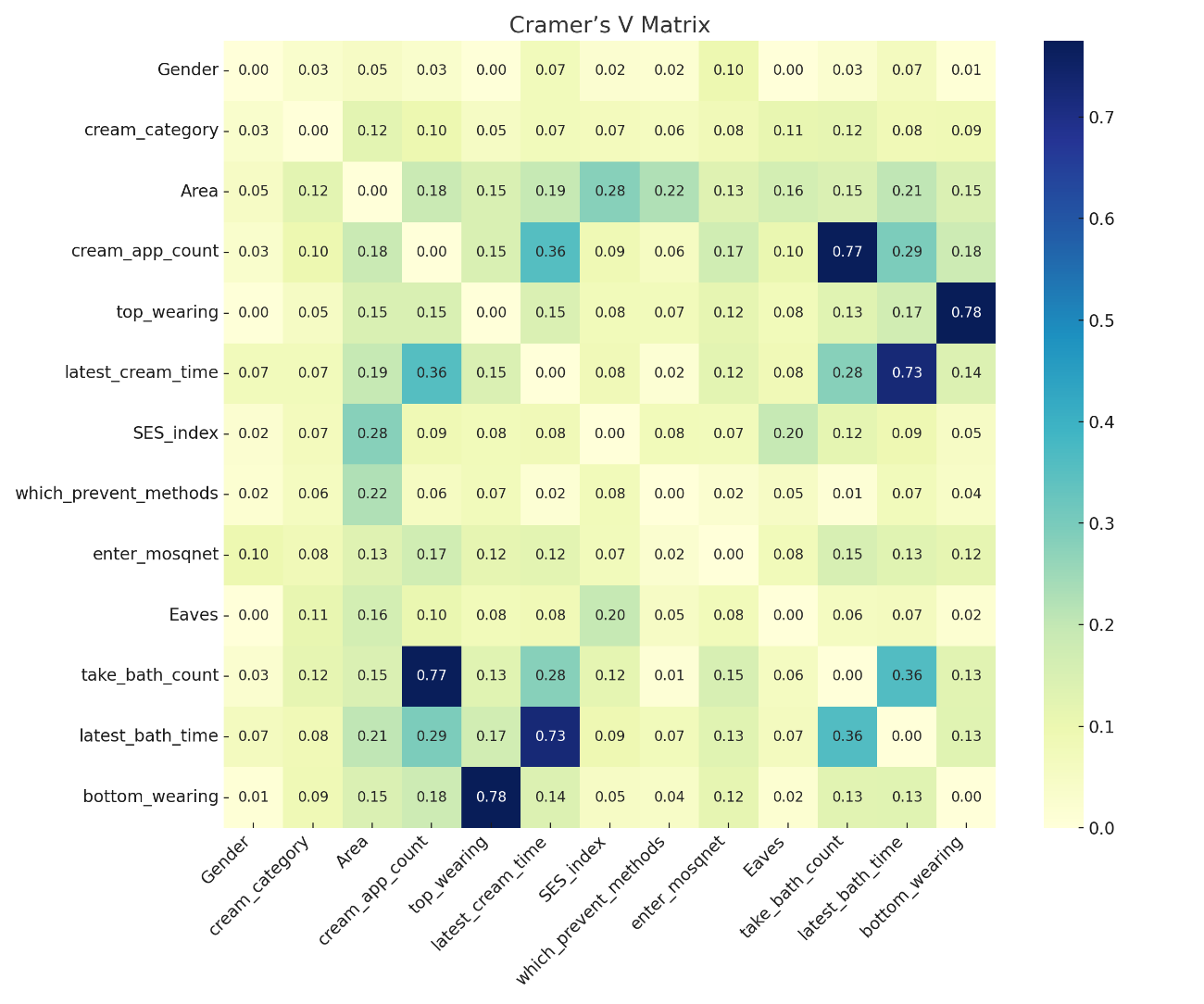


**Appendix 3-a.** Ethical approval for field work, Mount Kenya university


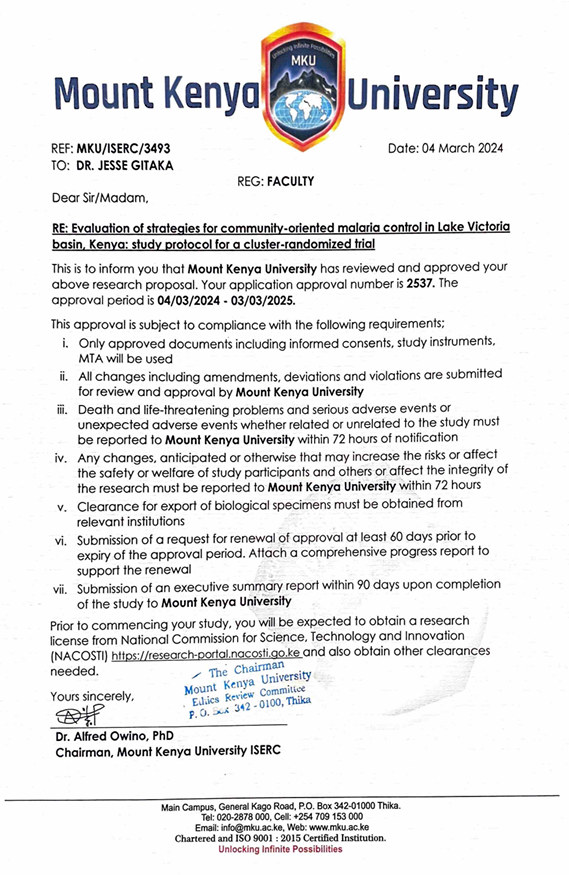


Appendix 3-b. Ethical approval for field work, Nagasaki university


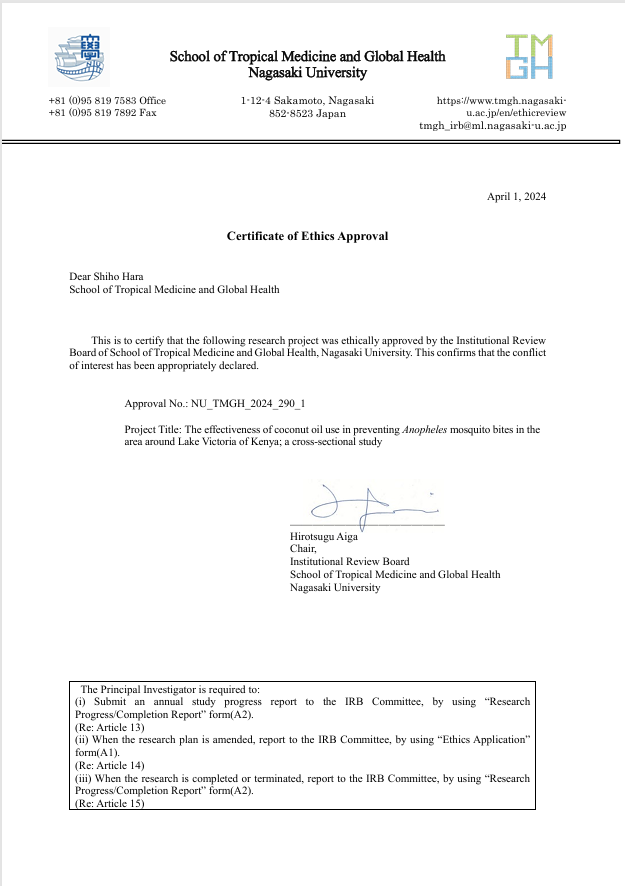


**Appendix 4.** Opt-out letter for establishing of mosquito bite definition

**
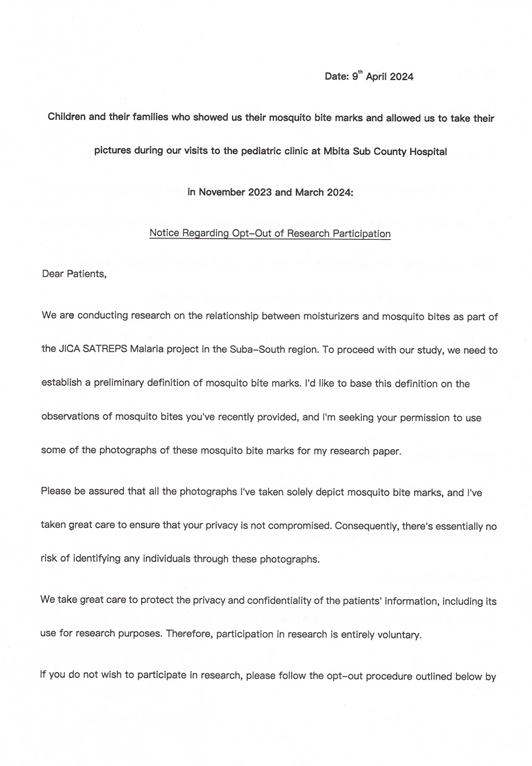
**

**
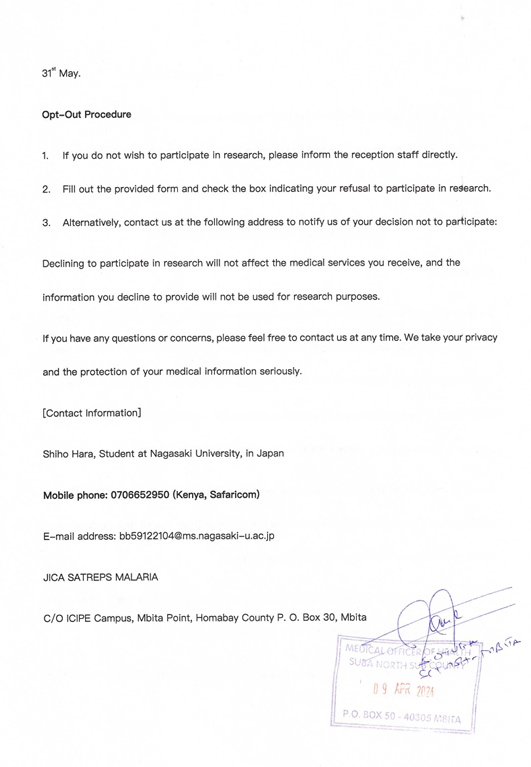
**

**Appendix 5**

**
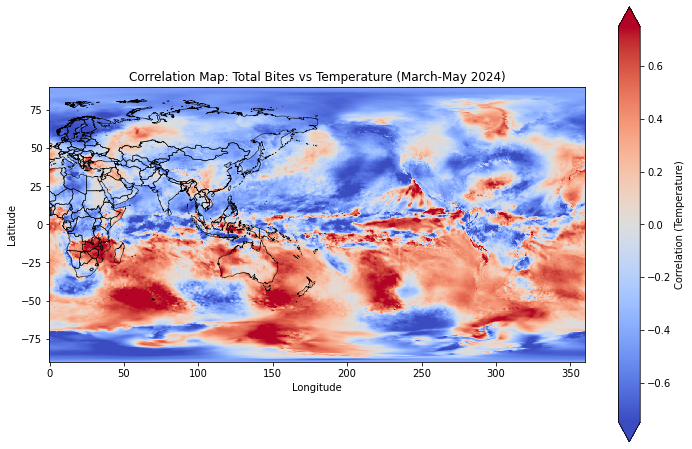
**
